# Predictability and variability of association patterns in sooty mangabeys

**DOI:** 10.1007/s00265-020-2829-y

**Published:** 2020-03-23

**Authors:** Alexander Mielke, Catherine Crockford, Roman M. Wittig

**Affiliations:** 1 Primate Models for Behavioural Evolution Lab, Institute for Cognitive and Evolutionary Anthropology, Oxford, UK; 2 Department of Psychology, University of Portsmouth, Portsmouth, UK; 3 Department of Primatology, Max Planck Institute for Evolutionary Anthropology, Leipzig, Germany; 4 Taï Chimpanzee Project, Centre Suisse de Recherches Scientifiques en Côte d’Ivoire, Abidjan, Côte d’Ivoire

**Keywords:** Sooty Mangabey, Entropy, Association, Social complexity, Social system, Fission fusion

## Abstract

**Abstract:**

In many group-living animal species, interactions take place in changing social environments, increasing the information processing necessary to optimize social decision-making. Communities with different levels of spatial and temporal cohesion should differ in the predictability of association patterns. While the focus in this context has been on primate species with high fission-fusion dynamics, little is known about the variability of association patterns in species with large groups and high temporal cohesion, where group size and the environment create unstable subgroups. Here, we use sooty mangabeys as a model species to test predictability on two levels: on the subgroup level and on the dyadic level. Our results show that the entirety of group members surrounding an individual is close to random in sooty mangabeys; making it unlikely that individuals can predict the exact composition of bystanders for any interaction. At the same time, we found predictable dyadic associations based on assortative mixing by age, kinship, reproductive state in females, and dominance rank; potentially providing individuals with the ability to partially predict which dyads can be usually found together. These results indicate that animals living in large cohesive groups face different challenges from those with high fission-fusion dynamics, by having to adapt to fast-changing social contexts, while unable to predict who will be close-by in future interactions. At the same time, entropy measures on their own are unable to capture the predictability of association patterns in these groups.

**Significance statement:**

While the challenges created by high fission-fusion dynamics in animal social systems and their impact on the evolution of cognitive abilities are relatively well understood, many species live in large groups without clear spatio-temporal subgrouping. Nonetheless, they show remarkable abilities in considering their immediate social environment when making social decisions. Measures of entropy of association patterns have recently been proposed to measure social complexity across species. Here, we evaluate suggested entropy measures in sooty mangabeys. The high entropy of their association patterns would indicate that subgroup composition is largely random, not allowing individuals to prepare for future social environments. However, the existence of strong assortativity on the dyadic level indicates that individuals can still partially predict who will be around whom, even if the overall audience composition might be unclear. Entropy alone, therefore, captures social complexity incompletely, especially in species facing fast-changing social environments.

**Electronic supplementary material:**

The online version of this article (10.1007/s00265-020-2829-y) contains supplementary material, which is available to authorized users.

## Introduction

Interactions between individuals in social animals do not take place in a social vacuum but are influenced by others in numerous ways. If bystanders influence the course and outcomes of interactions, then changing social environments has the potential to dramatically increase the information processing involved in animal decision-making (Aureli et al. 2008). If social complexity is defined by the amount of information necessary for an individual within a system to predict their own interaction patterns and those of other group members (Sambrook and Whiten 1997), then the flexibility of the social environment is an important measure of social complexity (Ramos-Fernandez et al. 2018). It should influence the evolution of cognition in multiple ways: if the audience of interactions is highly stable, then every interaction takes place under similar conditions. If the social environment is highly variable, every decision has to be made under different conditions (Aureli et al. 2008). Thus, understanding the variability and predictability of association patterns becomes fundamental in understanding the complexity of social decision-making in a species (Hemelrijk 1999; Whitehead 2008a; Ramos-Fernandez et al. 2018).

Bystanders have been shown to use information gleaned from observed interactions to assess mating partner quality (*Betta splendens*: Doutrelant and McGregor 2000) mating availability (baboons: Crockford et al. 2007), dominance rank (*Pinyon jays*: Guillermo Paz-Y-Miño et al. 2004), willingness to cooperate (*cleaner fish*: Bshary and Grutter 2006), and to decide whether to intervene into aggressive (rhesus macaques: Flack et al. 2006; chimpanzees: Preis et al. 2018a) and affiliative interactions (ravens: Massen et al. 2014; sooty mangabeys and chimpanzees: Mielke et al. 2017; mandrills: Schino and Lasio 2018; horses: VanDierendonck et al. 2009). Under these circumstances, species should evolve mechanisms that allow individuals to adapt their behaviour to the audience (Bshary and Noë 2003). Whether individuals decide to interact at all to signal their ability to cooperate (cleaner fish: Bshary and Grutter 2006; Pinto et al. 2011), change the form of interactions to increase the likelihood of being chosen as a partner (*Betta splendens*: Dzieweczynski et al. 2005 or adapt their partner choices to optimize their own fitness benefits (longtailed macaques: Gumert 2007; white-faced capuchins: Kajokaite et al. 2019; Barbary macaques: Kubenova et al. 2016; sooty mangabeys and chimpanzees: Mielke et al. 2018; Atlantic molly: Plath et al. 2008, 2009), the presence and composition of audiences can influence animal behaviour in important ways. There are increasingly detailed accounts of animals altering their behaviour in response to specific aspects of the relationships between potential interaction partners and observing bystanders (vervet monkeys: Borgeaud et al. 2017; baboons: Wittig et al. 2007; chimpanzees: Wittig et al. 2014; Kaburu and Newton-Fisher 2016; Mielke et al. 2018). Other examples of this flexibility in animal behaviour derive from studies of animal communication, where audience effects have been reported in various species (chimpanzees: Crockford et al. 2017; domestic chicken: Evans and Marler 1994; for reviews see: Zuberbühler 2008; Coppinger et al. 2017).

Species differences in spatial and temporal cohesion of groups and their fission-fusion dynamics have been argued to select for different socio-cognitive skills (Amici et al. 2008; Aureli et al. 2008). Primate species range from those with high spatial and temporal stability in who is close-by (all group members are constantly in visual contact with each other) to species where the overall group splits into distinct subgroups with strong variation in spatial cohesion, party size, and party composition (Aureli et al. 2008). In a recent paper, Ramos-Fernandez et al. (2018) proposed and tested the use of Shannon’s entropy as a measure for the temporal variation and predictability of subgrouping patterns. The article found higher levels of entropy in spider monkeys and chimpanzees, with high fission-fusion dynamics and high variability in the composition of subgroups than in geladas, who live in multi-level societies with highly stable one-male units, indicating higher social complexity in the former in this framework. However, in terms of information processing, complexity is traditionally thought of as an inverted U-shape (McShea 1996; Sambrook and Whiten 1997). Complexity is low if patterns are highly predictable; complexity is high when patterns are variable, but rules govern the system; and complexity is low again when the system is completely random. This should be mirrored in the entropy measures: if entropy is low, individuals can predict their social environment easily; for example, if all individuals are always close. If entropy is higher, but not indicating randomness (as in species with high fission-fusion dynamics), association patterns are variable but governed by underlying rules. If entropy is close to random, complexity from the perspective of the individual is low because no amount of information would allow for accurate predictions of the future state of the system (Sambrook and Whiten 1997). However, the question is how accurately entropy, which is focused on the composition of subgroups, can capture the predictability of who will be around from the perspective of individuals living in a primate social group.

In many primate species, individuals generally move cohesively (in that there are clear-cut fission events or there are no independently moving subgroups), but distributed over a large area to exploit resources efficiently. Thus, no clear lasting subgroups exist, but group size or environment makes it impossible for all group members to observe the interactions between all others. In spider monkeys, individuals seem to mainly influence each other when they are within visual range (Aureli et al. 2012). Thus, even though there is low variation in group-level spatial cohesion, the number and composition of group members in proximity and visibility might vary from the perspective of the individual (Farine et al. 2017). Amongst primates, this system is potentially represented by species with large multi-male, multi-female social groups. A system like this could have higher entropy than groups with high fission-fusion dynamics, such as in spider monkeys and chimpanzees if there is little overall consistency over time in the number or composition of individuals surrounding each group member.

The cognitive skills involved in navigating such a system should differ from those where individuals are in constant visual contact: like in high fission-fusion societies, individuals could benefit from monitoring the immediate social environment and base decision-making on the available information (Amici et al. 2008; Borgeaud et al. 2017; Mielke et al. 2018). Indeed, bystander effects on interactions have been observed in species with large groups and incomplete visibility, e.g., sooty mangabeys (Range and Noë 2005; Mielke et al. 2017, 2018), vervet monkeys (Borgeaud et al. 2017; Borgeaud and Bshary 2018), and baboons (Cheney and Seyfarth 2007). However, if future association patterns are largely unpredictable, we would not expect the evolution of cognitive skills that improve individuals’ ability to plan with whom they will be in spatial proximity, which might strongly influence with whom they can interact and who will observe each interaction (Amici et al. 2008). These skills might be of use in species with high fission-fusion dynamics, where individuals choose to associate in discrete parties (spider monkeys: Ramos-Fernández and Morales 2014; Busia et al. 2017; dolphins: Pearson 2009; Carter et al. 2013; and hyenas: Smith et al. 2007).

While individuals might not be able to predict all group members who will be present in their social environment, dyadic association preferences could lead to individuals being reliably surrounded by specific group members. Predictability of the social environment might be considerably higher than expressed by entropy measures if the presence of one group member reliably indicates the presence of another, even though who else is around is different at every time point. If entropy is low, then dyadic association patterns should be highly predictable as well. High entropy, on the other hand, might indicate that all group members associate almost randomly with each other. Alternatively, if the presence of one individual can be reliably used to predict the presence of a subset of others (e.g. if matrilines preferentially associate or high-ranking individuals monopolize resources together), then individuals might use this to imperfectly predict future association patterns: If I am with A, and A is usually associated with B, then it is likely that B will join us shortly. Even if it might still vary who else will be there with us, the predictability of the future state of the system is increased by these heuristic rules. In primates, individuals could spend all of their time with kin or same-sex individuals, but these clusters overlap with other clusters (e.g. other matrilines). Basic social categories might reduce the amount of information necessary when navigating social life. Here, we focus on how much basic parameters such as sex, kinship, rank, age, and reproductive state allow group members to predict which individuals will be close to each other because these parameters have been shown time and again to influence social structure.

Dyadic association could be structured along with several basic variables. Assortative mixing of individuals with similar attributes has been reported for a large number of species (Whitehead 2008a; Wey and Blumstein 2010; Madden et al. 2011; Carter et al. 2013; Hirsch et al. 2013), probably as the result of similar energetic (Muroyama 2017) and defensive interests (Tkaczynski et al. 2014; Josephs et al. 2016). Thus, we combined two kinds of analyses here: on one hand, we test sooty mangabeys subgroup entropy, assessing the variability of the social environment in which interactions take place from a structural level (Ramos-Fernandez et al. 2018). This analysis takes the identity of all subgroup members into account and represents the amount of information an individual would need to predict all possible combinations of group members on a certain spatial scale. On the other hand, we test whether on a dyadic level, association patterns are predictable, allowing incomplete but potentially sufficient prediction of future socio-spatial association and limiting the variability in social contexts under which interaction decisions are made. This analysis focuses on each dyad, not the association of the subgroup as a whole, and captures underlying rules that reduce the amount of variability in association patterns. Here, we characterize the predictability of association patterns in sooty mangabeys (*Cercocebus atys atys*) as a model for species with low fission-fusion dynamics living in large groups (Range and Noë 2002; Mielke et al. 2017, 2018). Sooty mangabeys are almost exclusively terrestrial. Visibility in their territory is usually around 10–30 m. The group will move around their territory in a relatively unconstrained way, with group members generally moving in a similar direction and no clear fission events. Interindividual distances vary depending on whether they are in a food patch, and the group can at times be stretched over several hundred square meters (AM, personal observation). Individuals can sometimes pass through the entire group in a very short time to get to a resource (AM, personal observation). A subgroup here is therefore defined from the perspective of the individual: an individual’s subgroup is all individuals who are in visual contact with them and could potentially observe interactions or become interaction partners. While little has been published on sooty mangabey association patterns, stable dyadic association patterns can be inferred from previous research. Sooty mangabeys show a proximity bias towards kin for juvenile and adult kin dyads (Range 2006). They have very stable hierarchies and show contest competition in food patches (Range and Noë 2002; C. Gba et al. unpublished data), indicating that high-ranking group members could potentially cluster in the centre of the group to monopolize food patches (Gba et al. 2019). However, while competition is most intense in fruit patches, mangabeys often rely on evenly distributed food sources, such as *Sacoglottis gabonensis* (McGraw et al. 2014), in which case group members can be spread over an area of several hundred square meters with limited visibility. Females and males, and females with young infants, sometimes associate for increased protection towards out-group males, who can join the group for days or months at a time (Fruteau et al. 2010). Adult males usually avoid each other and never groom each other (Mielke et al. 2018), but associate with females and subadult males. Grooming is directed towards females, individuals with similar rank, and regular cooperation partners (Mielke et al. 2018), as well as kin (Range 2006). We showed that while the composition of bystanders seems to vary constantly in sooty mangabeys, individuals take audience composition into account when making grooming decisions (Mielke et al. 2018): they were more likely to groom group members who did not have bond partners present and groomed closely-ranked group members when higher-ranking alternatives were absent. This is probably the case because bystanders intervene in both grooming and aggressive interactions in this species (Range and Noë 2005; Mielke et al. 2017), so adapting behaviour to bystander composition can potentially influence the outcome of an interaction. We predicted that group-level entropy in the mangabeys is very high, with association patterns close to random, given the large number of individuals and limited visibility in the forest environment. At the dyadic level, we predicted that mangabeys show dyadic partner preference, driven by kinship (Van Belle et al. 2014) and assortative mixing of individuals with similar dominance rank (Murray et al. 2007; Smith et al. 2007; Naud et al. 2016), age (Wey and Blumstein 2010; Heesen et al. 2015), sex (Heesen et al. 2015; Surbeck et al. 2017), and reproductive state (Collins 1984; Cowlishaw 1999).

## Methods

### Data collection

Mangabey data were collected in Taï National Park, Côte d’Ivoire (Boesch and Boesch-Achermann 2000) from October 2014 to October 2017. It was not possible to record data blind because our study involved focal animals in the field. All individuals were habituated to human presence, allowing close-range observation. Group size differed between years (2014/5: 40 individuals above 3 years—21 females, 19 males; 2015/6: 29 individuals above 3 years —20 females, 9 males; 2016/7: 28 individuals above 3 years—24 females, 3 males). We used 1-h continuous focal animal sampling (Altmann 1974). Although mangabey groups do not form clearly delineated subgroups, individuals do not see everybody all the time due to forest habitat caused visibility limitations. Therefore, subgroup composition was defined as group members in the visibility of observers following focal animals, with the assumption that these would also constitute the group members visible to the focal individual. Visibility in the Taï National Park is usually below 30 m. We recorded subgroup composition as scan samples every 15 mins by trained field assistants and researchers, and all group members that were visible to the observer, who watches the focal animal, were noted. This diverges from the definition of a subgroup chosen by some studies, which often rely on a ‘chain rule’, where individuals are considered part of a subgroup if they are within a specific distance of at least one other subgroup member (Aureli et al. 2012). However, under this rule, all group members would make up each subgroup in sooty mangabeys. Individuals in primate groups are probably mainly influenced by those group members in the visible range (Aureli et al. 2012). Using visibility might underestimate the number of individuals around the focal individual, but (a) it is the most parsimonious and replicable method for scan samples, (b) it is most comparable across species and field sites, and (c) there is no indication that there would be a directional bias introduced towards more random or more structured ‘subgroups. While the visible distance is thus lower than used in other species (Ramos-Fernandez et al. 2018), reducing the possible subgroup size, the entropy and association measures we use are based on expectations for subgroups of the observed sizes. Each scan was represented in the dataset by the presence (1) or absence (0) of each group member. On average, six individuals were observed together in a ‘subgroup’, with a maximum of 17 individuals. Around 87% of all subgroups included at least one male (average: two males) and around 97% of subgroups had at least one female (average: four females). The dataset was split into three yearly datasets lasting from October (beginning of birthing season) to September (end of mating season), thus encompassing both an entire mating and birthing season for each year. In this analysis, we included all group members that were above 3 years of age at the beginning of each year, because these are weaned and make movement decisions autonomous of their mother (Range 2006). Individuals were excluded if they were not part of the group the entire year (due to death or migration). For the 2014/5 season, we collected 4615 subgroup compositions; for 2015/6, we collected 5212 subgroup compositions; and for 2016/7, we collected 6601 subgroup compositions. To test whether the number of subgroup compositions was sufficient to reliably determine dyadic associations patterns for such a large group (Whitehead 2008b; Farine and Strandburg-Peshkin 2015), we bootstrapped the subgroup compositions for each year (1000 replicates) with replacement and calculated the standard deviations of dyadic simple ratio indices (Whitehead 2008b). The three seasons did not differ from each other, and all dyads had small standard deviations (maximum SD = 0.01), indicating that the sample size was sufficient to accurately predict association patterns in our group.

### Randomizations

As both the observed entropy and the dyadic association are compared to expected values, we used the same procedure to generate randomized datasets. We chose to use permutations to account for the underlying structure of our focal follow data collection. To construct appropriate null models and account for non-randomness of observation data (Farine 2013), we randomized group membership while keeping the party size (Ramos-Fernandez et al. 2018) and the identity of the focal individual in each scan constant (Surbeck et al. 2017). We also accounted for individual gregariousness by adjusting the likelihood that an individual was randomly selected in each scan by their occurrence likelihood in the observed data (Farine 2013). To account for autocorrelation between consecutive observations (Whitehead 2008a; Surbeck et al. 2017), consecutive scans that were the same in the observed data were also the same in the randomized data. This is a departure from the analytical bootstrap entropy proposed in Ramos-Fernandez et al. (2018), which does not account for focal follows and temporal autocorrelation. Based on these rules, we generated 1000 permutations of the observed scan data for each year, calculated the expected entropy and association measures, and compared these to the observed entropy and association measures.

### Entropy

To establish group-level entropy measures, we followed the script and guidance provided by Ramos-Fernandez et al. (2018). The entropy measure is based on the entire subgroup at each scan. Shannon’s entropy (Shannon 1948) or information entropy in this context is a measure for the amount of new information the observation of a specific party composition carries, compared to all other party compositions. If all subgroup compositions contained the same individuals, entropy would be minimal, as each single observation would not carry any information different from what is already available. If all subgroup compositions are different from each other, entropy would be maximal, as previous information would not allow the correct prediction of future subgroup compositions. Ramos-Fernandez et al. (2018) suggested the use of the Kullback-Leibler divergence (KL) and Jensen-Shannon distance (JS) as standardized measures of the distance between observed entropy and expected entropy that can be applied across species. However, when using data simulations to test how these indices deal with increasing group size and the relatively low data density usually used in primatological studies (see supplementary material), we observed that both measures are affected by group size and data density, and might, therefore, be unreliable when comparing different datasets. We, therefore, focus here on the direct comparison between the observed and predicted entropy (Ramos-Fernandez et al. 2018), calculated based on permutations of the observed data (described above). As a comparative measure across years, we used the ratio of the two entropy values (observed/predicted entropy; Batty et al. 2014). The ratio was not affected by data density or group size in the simulations, and could successfully differentiate between datasets with different levels of uncertainty.

### Dyadic association

To test whether dyadic association could potentially be a source of predictability in species with large groups who travel cohesively, we investigated whether there were preferred social partners in mangabey association patterns, and what determined preferred association. To this end, based on the same dataset as the group-level entropy, we calculated the observed simple ratio index (SRI_obs_) for each dyad for each year. The SRI was calculated as SRI=PAB/(PA + PB + PAB) (Farine and Whitehead 2015), where PAB is the number of times A and B were seen together, PA is the number of times A was seen, and PB is the number of times B was seen. We used the SRI despite the risk of individual identification errors because data were collected using focal follows and no robust calibration data existed (Hoppitt and Farine 2018). We compared the observed SRI with the expected SRI (SRI_exp_) for each dyad based on 1000 randomisations. We based our analysis on two measures of preferred association: first, on the pairwise affinity value (PAV), calculated by subtracting the observed from the predicted dyadic association (Surbeck et al. 2017). The resulting values were distributed between − 1 (dyad spends as little time together as possible) and 1 (dyad spends as much time together as possible). Dyads with a PAV above 0 associated more than expected, dyads with a PAV below 0 associated less than expected. Second, in addition to using a continuous measure, we tested which dyads had association values that were significantly higher than expected (Bejder et al. 1998). Including this binary approach increased the comparability of our study with previous results using an unweighted social network. A dyad was considered to be significant associates if SRI_obs_ was higher than 950 out of 1000 randomisations, corresponding with an alpha value of 0.05 and indicating that relationships between individuals are considered meaningfully if there is less than 5% chance that their association index is the result of random processes in the group.

### Factors determining the preferred association

To test whether there were factors that could increase the predictability of dyadic association, we investigated the impact of kinship, age, sex combination, rank difference, and the presence of new-born infants on the PAV and significant association patterns. We combined the dyadic values for each of the three years into one dataset. Dominance ranks were calculated using a modification of the Elo rating method (Foerster et al. 2016; Mielke et al. 2017) using non-aggressive feeding supplants in sooty mangabeys (Range and Noë 2002; Mielke et al. 2017). Ordinal ranks were standardized daily between 0 and 1. As individual rank values were stable and only changed due to demographic changes such as death and migration event, we used the average rank value for each individual for each year. The rank difference was the absolute difference between the ranks of the two individuals in the dyad in that year. In the models, we included the interaction between sex combination (male-male, female-male, female-female) and rank difference, as we would predict that females of similar rank associate preferentially, while males of similar rank avoided each other to prevent aggressive interactions with an unpredictable outcome. Kinship was binary, with genetically determined mother-offspring pairs and maternal siblings considered kin (AM et al., unpublished data). We categorized individuals as either adult (females above 5 years of age at the beginning of the year, males above 7 years of age) or subadult (above 3 years of age), resulting in adult-adult, adult-subadult, and subadult-subadult dyads. We entered the age combination into the model in interaction with sex combination, as differences between sexes in association patterns are likely to emerge over maturation (Range 2006). Mangabeys are seasonal breeders, with births clustering around January of each year. Mothers of new-born infants receive increased grooming (Fruteau et al. 2011; Mielke et al. 2018) and have been shown to be more central to the group in other primate species (Collins 1984; Cowlishaw 1999; Heesen et al. 2015). We classified dyads as either both, only one, or neither individual having infants in a year.

We fitted a Generalised Linear Mixed Model (GLMM) with binomial error structure to test which dyads were significant associates, and a Linear Mixed Model (LMM) with Gaussian error structure to test which predictors influenced the PAV, with each dyad represented for each year both individuals were present in the group (Baayen 2008). We include the sex combination–rank difference interaction, the sex combination–age combination interaction, kinship, and presence of infants as test predictors. We accounted for non-independent sampling by including the identities of both individuals, the identity of the dyad, and the year as random effects. To keep the type I error rate at the nominal level of 0.05 (Schielzeth and Forstmeier 2009; Barr et al. 2013), we included the random slopes for the rank difference in individual identities and year into the model. All models were implemented using the “lme4” package in R (Bates et al. 2015). We report the effect sizes of the models, calculated using the MuMIn package in R (Barton 2018), as quantification of the variance that the fixed and random effects explain and thus as ‘predictability’ of the dyadic association indices.

We used multimodel inference (Burnham and Anderson 2002) to test the relative impact each of the parameters had on whether two individuals preferentially associated. The set of models we fitted comprised all possible subsets of parameters of the full model. When interaction terms were included in a submodel, the two main effects were also included. The random effects structure was the same across all submodels. We determined the AICc (Akaike’s Information Criterion, corrected for small sample sizes; Burnham and Anderson 2002) for each submodel and determined Akaike weights and the 95% best model confidence set (Burnham and Anderson 2002). If the null model was not in the confidence set, we calculated the summed Akaike weight per predictor. These values were compared with the expected sum of weights assuming that all models performed equally well (Wessling et al. 2018).

The assignment the two individuals in a dyad as individual 1 and individual 2 is problematic for non-directed, dyadic measures such as association, as the random effects structure still impacts the variance explained by the fixed effects, making it necessary to account for the impact of the identities on the results (Kulik et al. 2012). We used repeated random selection of either one or the other individual as “individual 1” or “individual 2” to represent the dyad (Kulik et al. 2012; Mielke et al. 2017). We ran 1000 selections. For each selection, we fitted the full models to estimate the explained variance and repeated the multimodel inference approach for both the binomial and Gaussian model. We report the mean summed Akaike weights per predictor and effect sizes across the 1000 models as the results for the selections.

To test for multicollinearity, we used the function VIF of the R-package “car” (Fox and Weisberg 2011) to derive Variance Inflation Factors (VIF) (Field et al. 2012), applied to a standard linear model excluding the random effects and the interactions for each of the models. Collinearity was not an issue (maximum VIF = 1.98). The Linear Model with Gaussian error structure showed normal distribution of residuals. We tested for the presence of influential cases by systematically removing levels of the random effects (Field et al. 2012), which revealed no influential cases.

## Results

### Entropy

Comparing the observed and expected entropy values in sooty mangabeys shows a high level of entropy across all 3 years. In contrast to previous results for other species, mangabeys association patterns from 15 min scans were effectively random (2014/5: observed entropy: 11.83, expected entropy: 11.84; 2015/6: observed entropy: 11.99, expected entropy: 12.00; 2015/6: observed entropy: 12.36, expected entropy: 12.37), leading to the ratio of expected/observed entropy = 1 for all years. The almost random distribution of mangabeys association patterns is reflected in the number of unique subgroup compositions: in 2014/5, 4270 out of 4615 subgroup compositions (93%) were unique; 4630 out of 5212 subgroup compositions (89%) in 2015/6; and 6194 out of 6601 subgroup compositions (94%) in 2016/7.

### Dyadic association

**Fig. 1:**
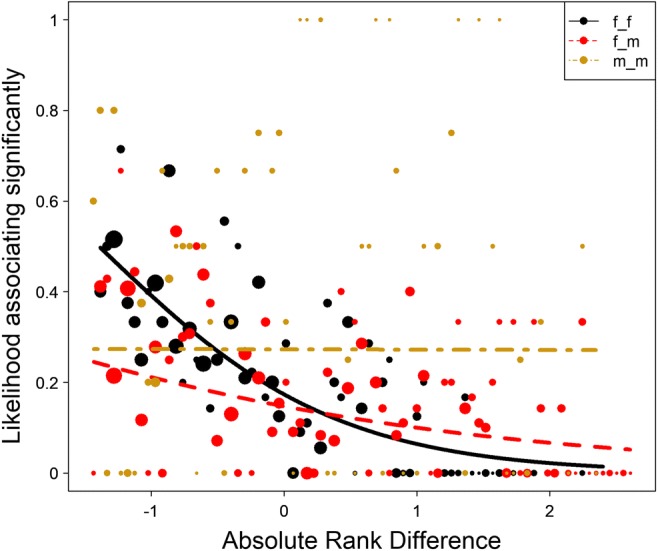
The probability of dyads associating with each other significantly depended on the effect of the interaction of absolute rank difference (z-standardized, original mean = 0.35, SD = 0.2) and the sex combination of the dyad. Points represent the likelihood that dyads are associated significantly more than expected (larger point volumes [range 1 to 24 observations] denote a larger number of observations), while lines represent the model results

**Fig. 2:**
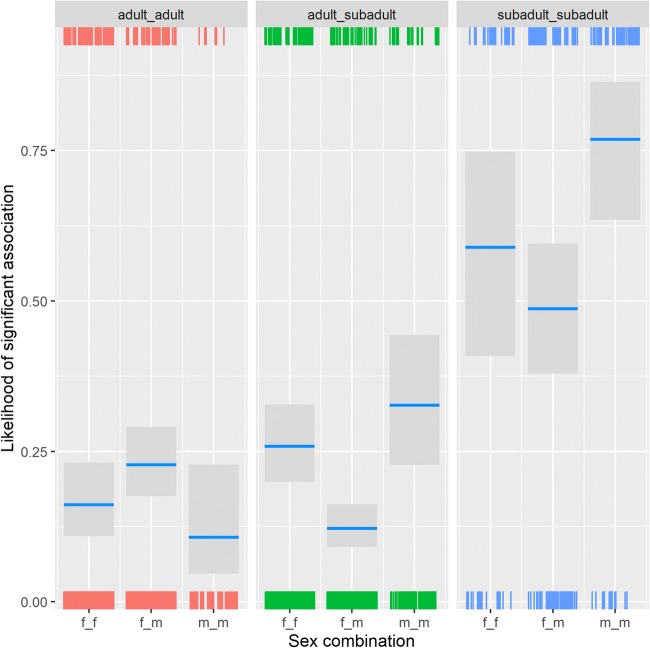
The probability of a dyad associating with each other significantly depending on the effect of the interaction of the age and sex combination of the dyad. The three sex combinations are shown separately for each age combination. Shown are the model result (lines), confidence intervals (grey block), and number of cases

**Fig. 3:**
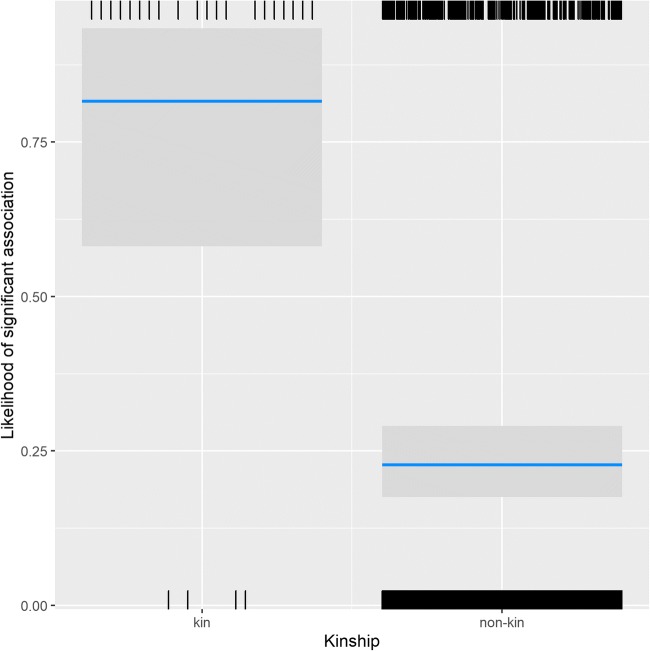
The probability of a dyad associating with each other significantly depending on the effect of kinship. Shown are the model result (lines), confidence intervals (grey block), and number of cases

**Fig. 4:**
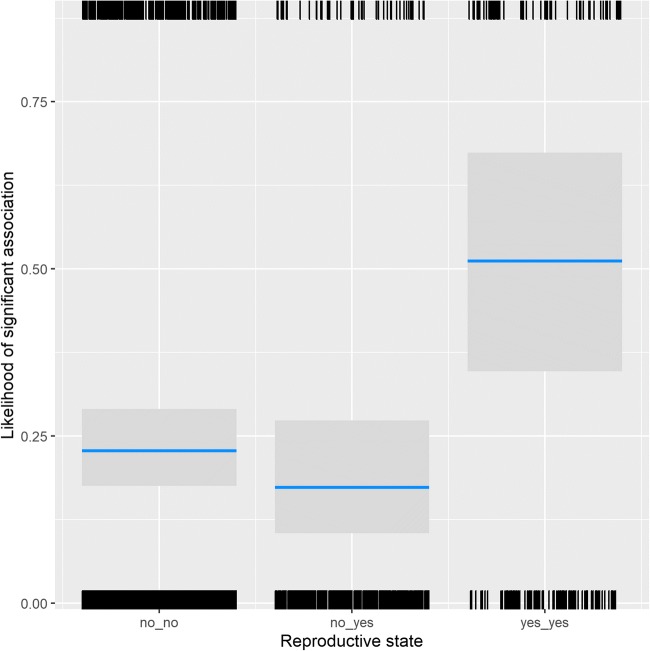
The probability of a dyad associating with each other significantly depending on the effect of the presence of new-born infants. S Shown are the model result (lines), confidence intervals (grey block), and number of cases

While association patterns were random when looking at all visible group members as a subgroup, clear and predictable patterns arose in dyadic associations, with assortativity of individuals based on kinship, rank, sex, and reproductive state. Both the Generalized Linear Mixed Model testing whether or not a dyad in a year was significantly more likely to be seen together than expected (χ^2^ = 217.02, df = 14, *p* < 0.001; marginal *R*^2^ = 0.28, conditional *R*^2^ = 0.55) and the Linear Mixed Model testing their pairwise affinity value (χ^2^ = 365.72, df = 14, *p* < 0.001; marginal *R*^2^ = 0.28, conditional *R*^2^ = 0.58) showed that the test parameters were significantly different in dyads that associated with each other. For both models, all predictors received strong support from the multimodel inference (Table 1). The interaction between sex combination and dominance rank difference showed that in male dyads, there was no effect of rank difference, while female and mixed dyads were more likely to be observed together if they were close in rank (Fig. 1). In both models, dyads of subadult individuals were much more likely to be associated than mixed-age dyads, who were in turn more likely to associate than adult dyads (Fig. 2). In subadult and mixed-age dyads, same-sex dyads were more likely to associate, while in adults, mixed-sex dyads were more likely to associate. Maternal kin was more likely to be observed together than non-kin (Fig. 3), and females with babies spent more time associated with each other than dyads containing only one female with an infant, or those not containing any females with infants (Fig. 4).

**Table 1:** Summary of the likelihood of dyadic association. Multimodel inference of the GLMM and LMM. Expected weights for each predictor variable are indicated in italics, and a sum of Akaike weights (based on AICc) per predictor that are larger than expected given the model set are indicated in bold. Estimates are the average estimates produced by the repeated random selection approach

		Significant association		Pairwise affinity value	
Term	Expected akaike weight	Estimate	Summed akaike weights	Estimate	Summed akaike weights
Kinship	*0.38*	2.4	**1**	0.39	**1**
Sex f_m^(1)^ * Rank Difference	*0.04*	0.76	**0.95**	0.05	**0.85**
Sex m_m^(1)^ * Rank Difference		1.12		0.08	
Sex f_m^(1)^ * Age adult_subadult^(2)^	*0.01*	− 1.41	**0.98**	−0.12	**1**
Sex m_m^(1)^ * Age adult_subadult^(2)^		− 0.11		0.09	
Sex f_m^(1)^ * Age subadult_subadult^(2)^		− 1.50		−0.20	
Sex m_m^(1)^ * Age subadult_subadult^(2)^		0.48		0.09	
Rank Difference	*0.40*	− 1.18	**1**	−0.10	**1**
Sex f_m^(1)^	*0.30*	− 0.12	**1**	0.05	**1**
Sex m_m^(1)^		0.61		−0.07	
Age adult_subadult^(2)^	*0.28*	0.61	**1**	0.10	**1**
Age subadult_subadult^(2)^		2.35		0.51	
Newborn Infants yes_no^(3)^	*0.27*	− 0.47	**1**	0.01	**1**
Newborn Infants yes_yes^(3)^		1.34		0.11	

(1) = reference level is female_female

(2) = reference level is adult_adult

(3) = reference level is no_no

## Discussion

In this study, we characterized the predictability of association patterns in sooty mangabeys. Social interactions in many animal species happen in changing social contexts, with bystanders actively and passively influencing decision-making and outcomes (Bonnie and Earley 2007; Mielke et al. 2017). Ostensibly, the variability of the audience influences the needs for flexible decision-making in social animals: if the audience is always the same, there is no need for individuals to optimize decision-making by taking the composition of the social environment into account (Aureli et al. 2008; Mielke et al. 2018). If the composition of the audience varies, individuals could gain benefits from adapting their choices flexibly to situational variables (Kaburu and Newton-Fisher 2015; Borgeaud et al. 2017) and from strategically altering their own movement patterns to create favourable conditions for future interactions (Amici et al. 2008). Here, we show that while sooty mangabey subgrouping patterns were highly random, on a dyadic level there were predictable patterns that could facilitate the prediction of the social environment. Thus, while individuals probably cannot predict the exact identity of all bystanders, easily heuristics might allow them to make a relatively accurate prediction about subsets of individuals that would be found together.

The use of the entropy of subgroup compositions has been shown to enable the differentiation between species with multi-level societies and those with high variability in party composition (Ramos-Fernandez et al. 2018). Here, we employed this measure using visibility scans of a species living in large multi-male, multi-female groups where group members move through their territory as a unit but are separated visually. Using data simulations (supplementary material), we showed that the ratio between the observed and expected entropy is a reliable comparative measure between animal groups of different sizes. The entropy differences indicated that knowing whether a set of group members were associated in the past does not allow to make statements about the likelihood that this same set of individuals will be associated in the future, as subgroups derived from scan samples were random. All group members of the sooty mangabey group usually move in the same general direction, but which food source they target on their way varies, and individuals can sometimes pass the entire group when they want to get to a particular resource (AM, personal observation). Mangabeys are thus unlikely to be able to predict exactly who will be close-by for future interactions, and each interaction takes place in a different social context from the last. From an information processing perspective as the basis for complexity (Sambrook and Whiten 1997), this would indicate that mangabey subgrouping is extremely simple because no amount of information would allow prediction of future composition.

While the exact composition of the social environment might be hard to predict for individuals, introducing a strong element of uncertainty into their social life, we found strong dyadic preferences of association. These two results are not mutually exclusive: For example, a matrilineal family group could spend all of its time in close proximity, but move through the forest in a way that crosses the path of a constantly changing array of other group members. From the subgroup perspective, this would mean that each observed subgroup is ‘unique’; given the size of many primate groups, the number of unique subgroups is vast. From the perspective of any individual, who is found together and who is a bystander of an interaction is probably much more predictable because there are simple heuristic rules that allow for imperfect predictions of who will be found together at any given time. Like other animal species, sooty mangabeys showed strong assortative mixing based on kinship (giraffes: Carter et al. 2013; black howler monkeys: Van Belle et al. 2014), age (assamese macaques: Heesen et al. 2015; yellow-bellied marmots: Wey and Blumstein 2010), dominance rank (chimpanzees: Murray et al. 2007; spotted hyena: Smith et al. 2007; vervet monkeys: Teichroeb et al. 2015), and the presence of new-born infants (yellow baboons: Collins 1984; chacma baboons: Cowlishaw 1999; assamese macaques: Heesen et al. 2015). This indicates that individuals clustered based on similar energetic needs (based on age and reproductive state), similar levels of power (with closely-ranked females clustering around valuable resources they can access), and tolerance for kin and the opposite sex in adults. Subadult individuals clustered strongly, especially same-sex dyads, while mixed-sex dyads were more likely to associate in adults. The latter could be the result of adult females and males staying in proximity to offer infant protection against outgroup males (Fruteau et al. 2010). Future studies should take into account the spatial position of individuals in the group, to test whether competition for resources and safety explain clustering of closely-ranked individuals, mothers with young infants, and subadults (Tkaczynski et al. 2014; Teichroeb et al. 2015), and which individual-level decision-making processes underlie emergent group-level patterns (Bonnell et al. 2017). Also, sooty mangabeys use contact vocalizations which could potentially widen the perceived subgroup composition as individuals know about the movements of group members who are out of sight (Range and Fischer 2004).

Assortativity provides important information that might increase predictability and reduce the number of different social contexts even in a system without clear subgrouping. Importantly, not every group member has an influence on interaction outcomes: in different primate species, the presence of high-ranking bystanders (vervet monkeys: Borgeaud et al. 2017; chimpanzees: Kaburu and Newton-Fisher 2016), potential female partners (longtailed macaques: Gumert 2007), and the social relationship of bystanders with possible interaction partners (sooty mangabeys and chimpanzees: Mielke et al. 2018) have been shown to impact decision-making. Also, certain bystanders are more likely than others to intervene into socio-positive (sooty mangabeys and chimpanzees: Mielke et al. 2017; stumptail macaques: Mondragón-Ceballos 2001; mandrills: Schino and Lasio 2018) and aggressive interactions (pigtail macaques: Castles et al. 1996; white-faced capuchins: Kajokaite et al. 2019; chimpanzees: Preis et al. 2018b, Wittig and Boesch 2003). Thus, the identity of all bystanders might be less informative than the presence of those relevant as interveners or observers, and simple heuristics might allow individuals to circumvent the need to monitor every group member present. If the presence of specific influential individuals has a strong impact on the social context in which interactions take place, then entropy alone will not be sufficient to map the variability and predictability facing individuals in animal social groups. Thus, the entropy measure might fail to capture social complexity accurately and overestimate the information content inherent in the variation of subgrouping patterns in primate groups. Entropy quantifies how complex a group is from a system-perspective, and gives a value to upper bounds of the problem primates face; it cannot account for the fact that individuals will use repeatable patterns to make best guesses. Because the entropy measure is based on the full subgroup composition, it fails to identify if parts of each subgroup are highly stable. For example, in a group with five individuals, the subgroup compositions 1/1/1/0/0, 1/1/0/1/0, and 1/1/0/0/1 are considered unique, but part of each subgroup is highly conserved. Thus, to understand the complexity of social association patterns of a group, both dyadic and entropy measures need to be considered in concert.

This raises questions about the challenges that movement patterns create in large social groups. Our results indicate that the variability in the social context, even given the predictability arising from assortative mixing, is considerably higher than that faced by species with clear subgroups which might persist for hours or days at a time (Aureli et al. 2008). Thus, the information processing capacity needed to keep track of the immediate social environment might actually be higher than in species with high fission-fusion dynamics, and individuals might need to be more flexible in reacting to new arrivals and departures. Mangabeys adapt their behaviour to the present social environment (Mielke et al. 2018), monitor interactions between others (Mielke et al. 2017), and influence those interactions through interventions (Range and Noë 2005), indicating that they possess the capacity to keep track of changing circumstances and use the presence and absence of others to their advantage. However, the variability would make it hard to predict who will be a bystander of an interaction at a given point in time or wait for the optimal party composition to engage in certain interactions, something that has been argued for species with high fission-fusion dynamics (Amici et al. 2008). Inhibition of interactions in anticipation of the ‘optimal’ social context, and mental time-travelling abilities, might have limited use for mangabeys (Aureli et al. 2008), as their ability to adapt their own movement patterns to create beneficial social situations is limited. Our results thus help shed a light on how differences in social organization, for example between species with and without spatially separated subgroups, could create different cognitive challenges for individuals living in those systems (Byrne and Whiten 1989).

## Electronic supplementary material

ESM 1 (DOCX 1.02 MB)

## Data Availability

All data are deposited in https://data.mendeley.com/datasets/xy6h7pxkz7/draft?a=337269d3-3ba0-451c-b705-60c8ca5dc92a
