# Supplementary material for: Predictability and variability of association patterns in sooty mangabeys
Source: Behav Ecol Sociobiol. 2020 Mar 23;74(4):46. doi: 10.1007/s00265-020-2829-y (PMC7089916; doi:10.1007/s00265-020-2829-y)
Supplement: Supplementary file 1 — (DOCX 1.02 MB) [file 265_2020_2829_MOESM1_ESM.docx]

***Behavioral Ecology and Sociobiology***

**Predictability and variability of association patterns in sooty mangabeys**

**Mielke, Alexander ^1,2,3,4^; Crockford, Catherine ^3,4,#^; Wittig, Roman M. ^3,4,#^**

1 Primate Models for Behavioural Evolution Lab, Institute for Cognitive and Evolutionary Anthropology, Oxford, UK

2 Department of Psychology, University of Portsmouth, UK

3 Max Planck Institute for Evolutionary Anthropology, Department of Primatology, Leipzig, Germany

4 Taï Chimpanzee Project, Centre Suisse de Recherches Scientifiques en Côte d'Ivoire, Abidjan, Côte d'Ivoire

^#^joint senior author

**Correspondence**

Alexander Mielke: [mielke.alexand@gmail.com](mailto:mielke.alexand@gmail.com)

**Supplementary Material**

**Simulation impact of data density and community size on entropy measures**

When introducing Shannon’s entropy as a measure for predictability in association patterns, Ramos-Fernandez et al. (2018) make use of three indices to compare groups of different sizes and different social systems: the comparison between observed entropy and predicted entropy given random distribution (by calculating the ratio of observed entropy/predicted entropy; this ratio should approach 0 for complete order, and 1 for complete randomness), the Kullback-Leibler (KL) divergence, and the Jensen-Shannon (JS) distance. To be useful for comparative research, indices have to be robust across datasets with different numbers of individuals and different data collection density, so we used data simulations to determine how each of these indices performed under different conditions.

We simulated two different conditions: in the first condition, we created very large datasets (10,000,000 rows) for different numbers of ‘group members’ (13-25 individuals). For each row, the number of present ‘individuals’ was randomly selected. After the number of individuals in a row was assigned, the actual individuals were also assigned randomly. Thus, the dataset should show complete randomness in the different entropy measures. We then reduced the dataset by selecting subsets of different sizes, to simulate different data collection densities (2000-15,000 data points, as these are dataset sizes that most animal studies will work with). We then applied the script kindly provided by Ramos-Fernandez et al. (2018) in their supplementary material to calculate the observed and predicted entropy, KL divergence, and JS distance for each dataset. We predicted that all three indices would accurately reflect the perfect randomness of the data, with the KL divergence and JS distance being close to 0, and the observed and expected entropy being the same. Importantly, the entropy measures should not be influenced by group size and data density; if they are, they do not lend themselves readily for between-group comparisons. In the second condition, we created a dataset consisting of 13-25 individuals and 10,000 rows, and simulated the impact of increasing order on the three indices. We randomly created a number of rows (between 0.02% and 100% of all rows) that where then replicated to reach 10,000 cases. Thus, in the extremely ordered case, the dataset was made up of 2 rows that were repeated over and over again. At the other end, data were again random. The KL divergence and JS distance should approach 1 with increasing order, and the observed and expected entropy should diverge and their ratio approach 0. For each iteration, we also calculated the measures for the randomized version of the dataset. This was done to see whether the JS distance and KL divergence of small random datasets actually approach 0.

The results of our simulations showed that the KL divergence and JS distance were influenced by data density and the number of individuals in the community. As shown in Fig. S1 and S2, despite complete randomness of the data, both indices measured increasing order with increasing number of individuals, independent from the sample size. If they were capturing only whether subgroup distribution was random, both values would be 0 for all datasets, but this is not the case for larger communities. Thus, comparing two primate groups with the same underlying structure but different amounts of data or individuals might nonetheless create different results, which is not useful for a comparative measure. Importantly, the same community could yield different results, depending on whether for example subadult individuals are included in the analysis or not. It is possible that for increasing group size, due to the larger number of possible combinations, more cases are needed to reliably detect whether the distribution of values was random; however, if this is the case, then these measures are not suitable for the sample sizes usually found in animal research, unless data are collected remotely. The same was not true for the ratio observed and expected entropy, which indicated randomness under all conditions, with only very weak decrease due to group size (Fig. S3).


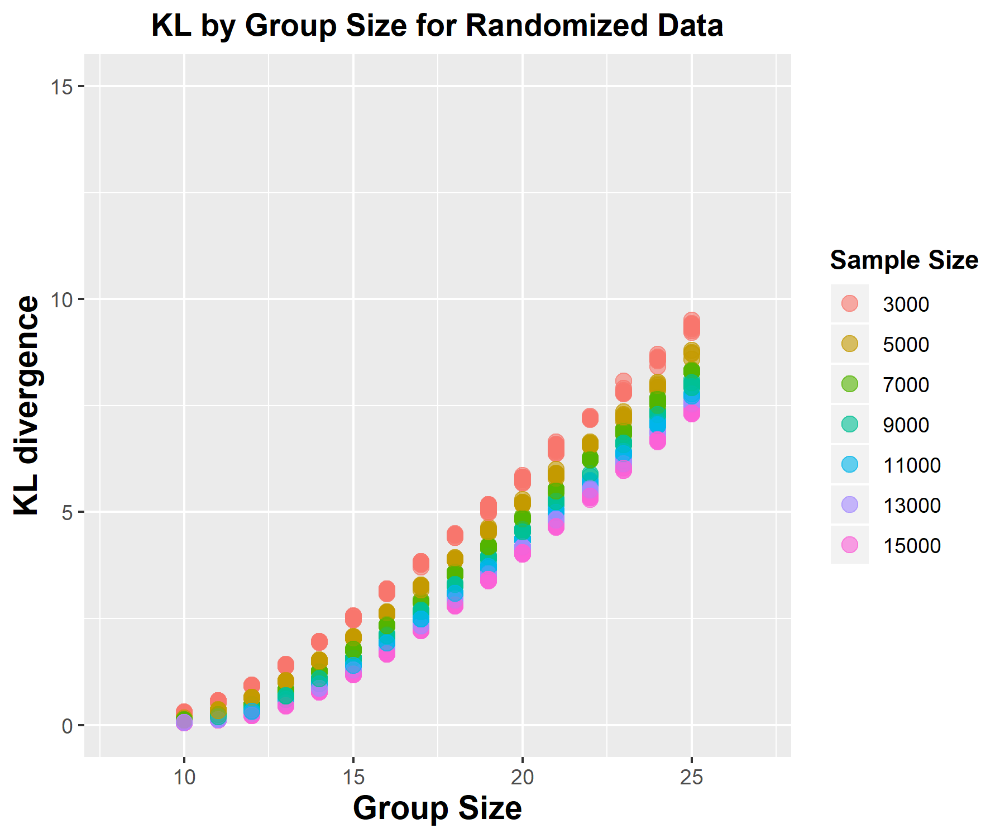


Figure S1: Kullback-Leiber divergence (KL) for perfectly random datasets for groups of different sizes and different amounts of data.


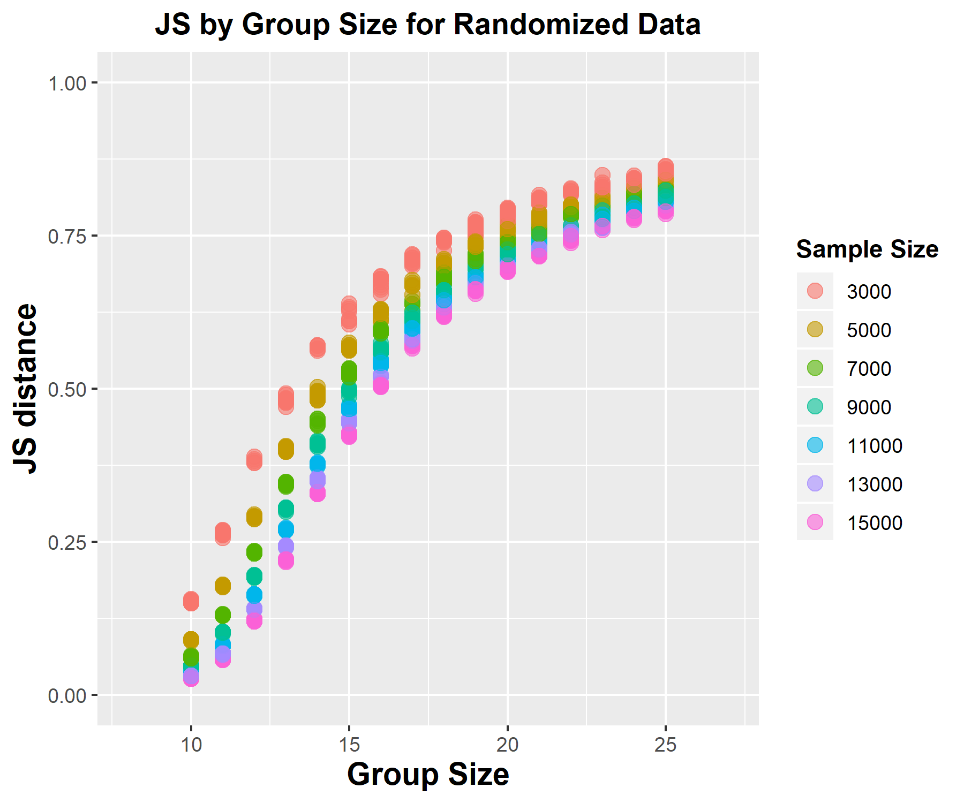


Figure S2: Jensen-Shannon distance (JS) for perfectly random datasets for groups of different sizes and different amounts of data.


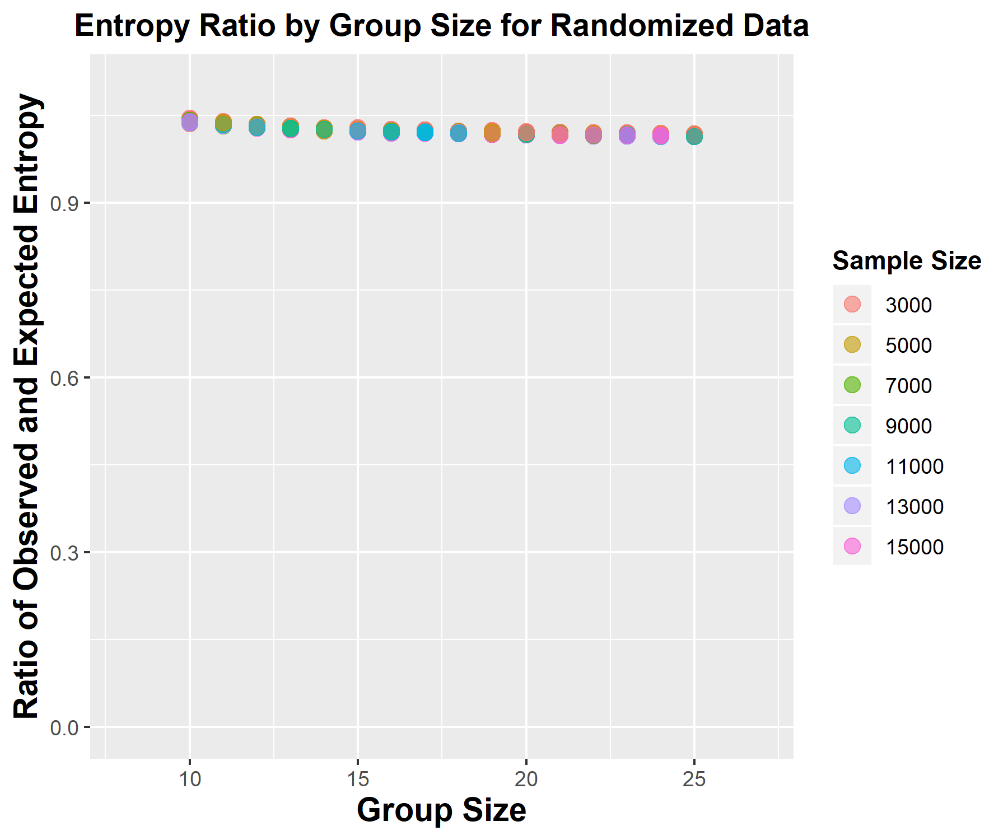


Figure S3: Ratio between observed and expected entropy values for perfectly random datasets for groups of different sizes and different amounts of data. High overlap between observed and expected values obscures unfilled squares.

The results of our simulations given increasing order showed that both the JS distance and KL divergence did pick up on differences in order in the data (Fig. S4 and S5). However, again, the values cannot be interpreted independent of group size: except for perfect order, the values for the same underlying rules differed dramatically depending on group size. The same JS distance (e.g., 0.6) could be generated by a completely random large group, or a highly ordered small group. Again, the ratio of the observed and expected entropy was the measure that most accurately described the expected patterns (Fig. S6).


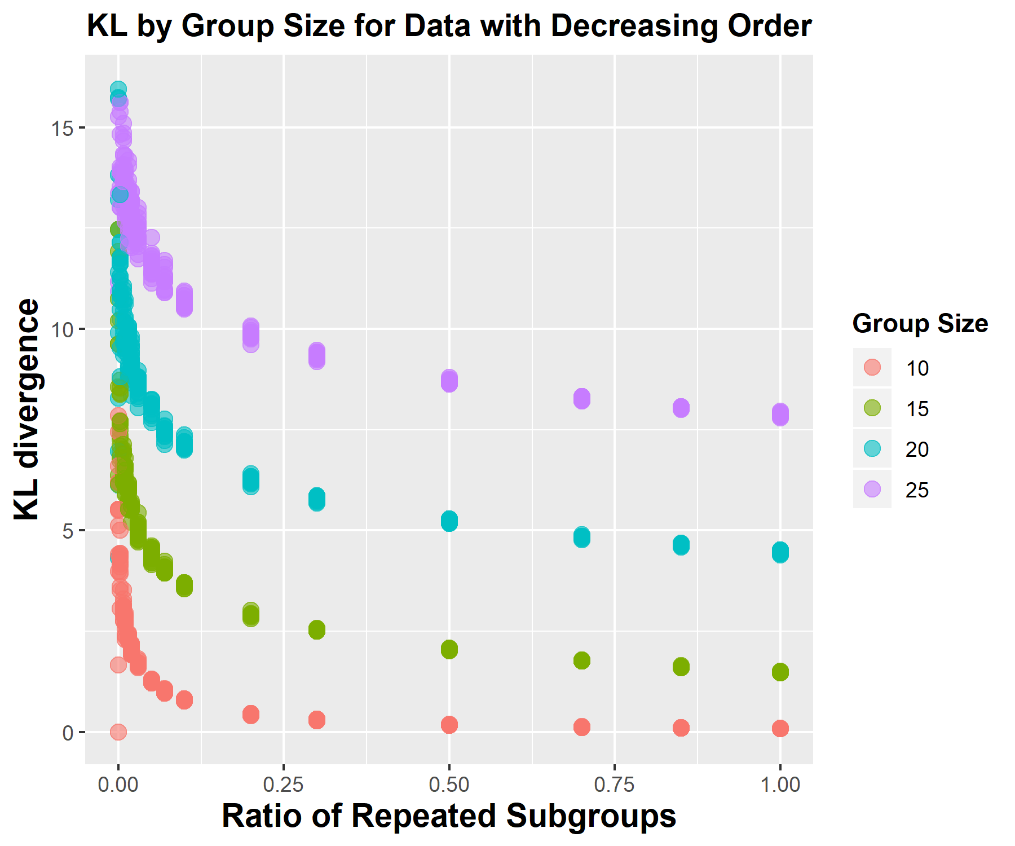


Figure S4: Kullback-Leiber divergence (KL) for datasets with decreasing levels of order (1 means all subgroups are unique; 0.0001 means that 1 out of 10,000 subgroups is unique) for groups of 10, 15, 20, and 25 individuals and 10,000 data points. KL divergence values for random data should be 0.


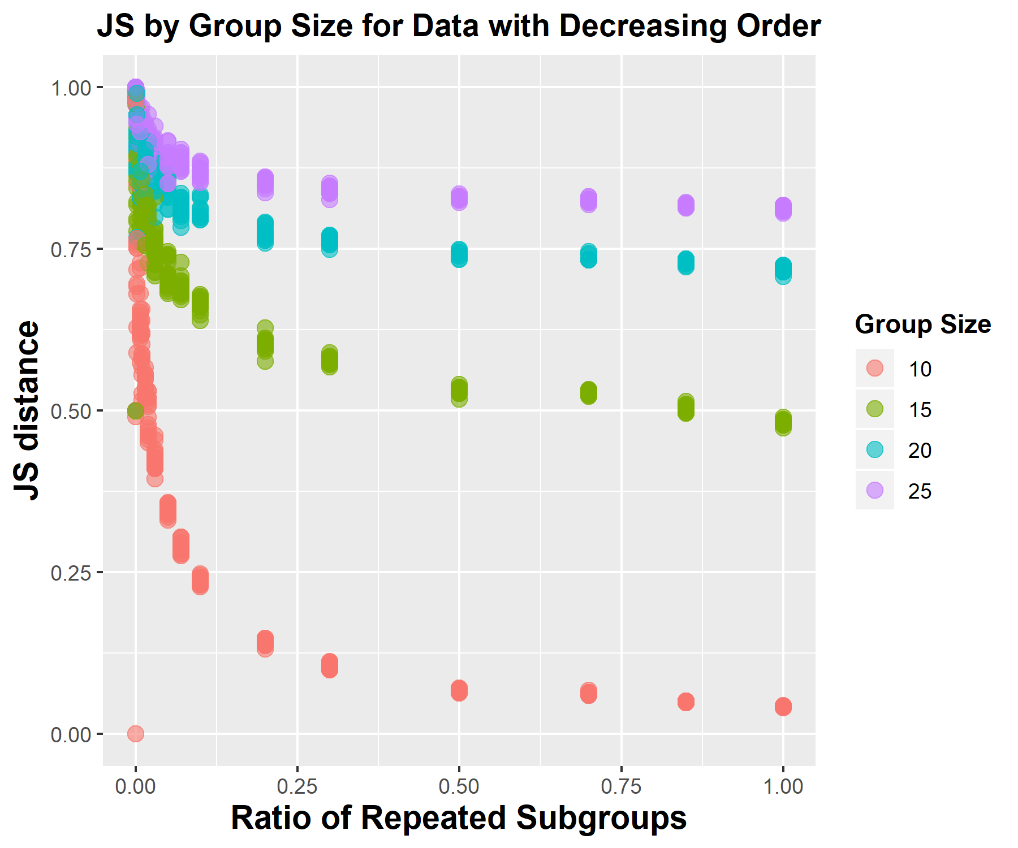


*Figure S5: Jensen-Shannon distance (JS) for datasets with decreasing levels of order (1 means all subgroups are unique; 0.0001 means that 1 out of 10,000 subgroups is unique) for groups of 10, 15, 20, and 25 individuals and 10,000 data points. JS distance values for random data should be 0.*


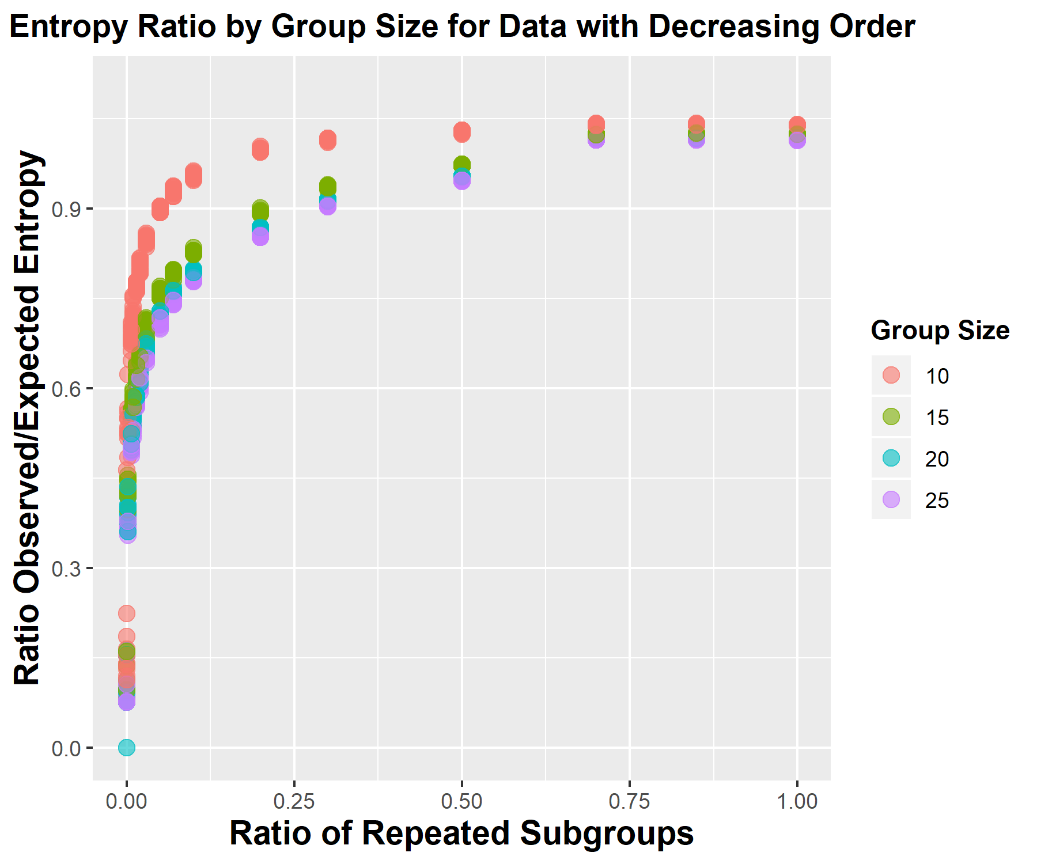


*Figure S6: Ratio between entropy values for datasets with decreasing levels of order (1 means all subgroups are unique; 0.0001 means that 1 out of 10,000 subgroups is unique) for groups of 10, 15, 20, and 25 individuals and 10,000 data points. The ratio of observed and expected entropy values for random data should be 1.*

Given these results, the ratio of observed entropy and expected entropy under random conditions was the most robust measure of entropy when comparing groups of different sizes. We would therefore recommend the use of the ratio between the two values: this ratio observed/expected should approach 1 if association patterns are highly randomized and unpredictable, while it should approach 0 if association patterns are highly ordered. The reason that the KL divergence and JS distance did not perform as expected seems to be the sheer number of possible combinations in large primate communities: groups of 30 individuals can associate in more than 1 billion different ways. It seems that the two measures interpret the fact that some combinations were not observed as a form of order. However, animal datasets are exceedingly small, so if only 10,000 data points exist, it is not clear whether the missing combinations cannot occur, or data were missing, as in the case of the simulations. The expected entropy takes this limitation into account, which is why the entropy ratio performs as expected. This is not to say that the JS distance and KL divergence cannot become useful measures in the future; however, their usage has to be adapted to the specific properties of animal association datasets.
